# Association of Toxicity of Sorafenib and Sunitinib for Human Keratinocytes with Inhibition of Signal Transduction and Activator of Transcription 3 (STAT3)

**DOI:** 10.1371/journal.pone.0102110

**Published:** 2014-07-11

**Authors:** Kazuhiro Yamamoto, Atsushi Mizumoto, Kohji Nishimura, Atsushi Uda, Akira Mukai, Kazuhiko Yamashita, Manabu Kume, Hiroo Makimoto, Toshinori Bito, Chikako Nishigori, Tsutomu Nakagawa, Takeshi Hirano, Midori Hirai

**Affiliations:** 1 Department of Pharmacy, Kobe University Hospital, Kobe, Japan; 2 Division of Pharmacokinetics, Kobe University Graduate School of Medicine, Kobe, Japan; 3 Division of Dermatology, Kobe University Graduate School of Medicine, Kobe, Japan; University of Tennessee, United States of America

## Abstract

Hand–foot skin reaction is a most common multi-kinase inhibitor-related adverse event. This study aimed to examine whether the toxicity of sorafenib and sunitinib for human keratinocytes was associated with inhibiting signal transduction and activator of transcription 3 (STAT3). We studied whether STAT3 activity affects sorafenib- and sunitinib-induced cell growth inhibition in HaCaT cells by WST-8 assay. Stattic enhanced the cell-growth inhibitory and apoptotic effects of sorafenib and sunitinib. HaCaT cells transfected with constitutively-active STAT3 (STAT3C) were resistant to the sorafenib- and sunitinib-induced cell growth inhibition. STAT3 activity decreased after short-term treatment with sorafenib and sunitinib in a dose-dependent manner and recovered after long-term treatment with sorafenib and sunitinib at low doses. Moreover, the expression of survivin and bcl-2 decreased after treatment with sorafenib and sunitinib was concomitant with variations in STAT3 activity. Sorafenib-induced STAT3 inhibition was mediated by regulation via MAPK pathways in HaCaT cells, while sunitinib-induced STAT3 inhibition was not. Thus, STAT3 activation mediating apoptosis suppressors may be a key factor in sorafenib and sunitinib-induced keratinocyte cytotoxicity.

## Introduction

Molecular-targeted drugs have lead to innovative progress in cancer chemotherapy. At present, although a reduction has been observed in the discovery of novel candidate therapeutic compounds, a novel target molecule for cancer therapy and compounds with particular affinity for this molecule have been developed in a study. A clinical trial for these compounds has been conducted for various types of cancer [1]. Sorafenib and sunitinib are the first oral multikinase inhibitors that target Raf-1 and receptor tyrosine kinases, including vascular endothelial growth factor receptors (VEGFRs), platelet-derived growth factor receptor (PDGFR), c-Kit, Flt-3, and RET [2], [3]. These have been used as first-line therapy for renal cell carcinoma (RCC) and hepatocellular carcinoma worldwide and have demonstrated favorable outcomes. Recently, axitinib and pazopanib have been included as drugs that function as multikinase inhibitors; hence, multikinase inhibitors play an important role in cancer chemotherapy [4], [5].

Although molecular-targeted therapy is considered to be more safe, it is associated with common problems in clinical practice. Skin-related side effects are observed for these drugs with exceptionally high frequency, including 48% with sorafenib therapy and 36% with sunitinib therapy [6], resulting in interrupted therapy or decreased quality of life. Although it is considered that these symptoms are apparently due to a diminished proliferative ability of keratinocytes, the biological mechanisms remain unclear.

Signal transducer and activator of transcription 3 (STAT3) is a point of convergence for numerous tyrosine kinases, including VEGFR, PDGFR, EGFR, and Src, among many others [7], [8]. STAT3 has a critical role in various biological activities, including cell proliferation, survival, and homeostasis through regulation of related genes including the inhibitors of apoptosis family [9]–[14]. STAT3 was the primary factor in the regulation of cutaneous homeostasis, as reported by a recent study [11], [15]. The dermatological adverse events induced by molecular-targeted therapy is potentially caused by a change in the activity of STAT3 as a primary factor in the progression of skin lesions.

In this study, we investigated the effects of STAT3 and related mechanisms on sorafenib- and sunitinib-induced cell growth inhibition in a human immortalized keratinocyte cell line. Our findings suggest that STAT3 activity in keratinocytes may be a key factor in sorafenib- and sunitinib-induced dermatological events.

## Materials and Methods

### Chemicals

**Figure 1:**
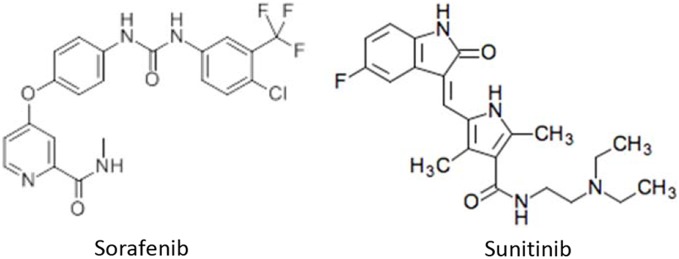
Chemical structures of sorafenib and sunitinib.

Sorafenib was purchased from LKT Laboratories, Inc. (St. Paul, MN, US). Sunitinib malate and Hoechst 33258 were purchased from Sigma-Aldrich Chemical, Co. (St Louis, MO, US). Chemical structures of sorafenib and sunitinib show Figure 1. Stattic, a small-molecule inhibitor of STAT3 activation [16], was purchased from Enzo Life Sciences, Inc. (Farmingdale, NY, US). SB203580 and U0126 were purchased from Cell Signaling Technology, Inc. (Boston, MA, US).

### Antibodies

Rabbit anti-phosphorylated (anti-phospho)-STAT3 at tyrosine 705 (Tyr705) and serine 727 (Ser727), rabbit anti-STAT3, rabbit anti-survivin, rabbit anti-Bcl-2, rabbit anti-Mcl-1, rabbit anti-β-actin, and anti-rabbit HRP-conjugated IgG were purchased from Cell Signaling Technology. Anti-rabbit fluorescein isothiocyanate (FITC)-conjugated IgG was purchased from Santa Cruz Biotechnology (Dallas, TX, US).

### Cells and cell culture

HaCaT cells, a human immortalized keratinocyte cell line, were kindly provided by Professor Norbert Fusenig (German Cancer Research Centre, Heidleberg, German) [17]. HepG2 cells, a human hepatocarcinoma cell line, were purchased from JCRB (Osaka, Japan). HaCaT and HepG2 cells were maintained in Dulbecco’s modified Eagle’s medium (Sigma-Aldrich) supplemented with 10% heat-inactivated fetal bovine serum (FBS; MP Biomedicals, Solon, OH, US) and antibiotics (Life Technologies Corporation, Carlsbad, CA, US). Caki-1 cells, a human renal carcinoma cell line, were obtained from JCRB. Caki-1 cells were maintained in RPMI-1640 medium (Sigma-Aldrich) supplemented with 10% heat-inactivated FBS and antibiotics.

### WST-8 colorimetric assay

The effects of various signal transduction inhibitors and transfection with a STAT3 construct on sorafenib-induced cell growth inhibition in each cell line were evaluated by WST-8 assay using a Cell Counting Kit-8 (Dojindo Laboratories, Kumamoto, Japan). Cells were seeded at a density of 1×10^3^ cells/well in 96-well plates and precultured for 24 h. Cells were either pretreated with signal transduction inhibitors at various concentrations for an appropriate period or transfected with a STAT3 plasmid (described below). Thereafter, the culture medium was replaced with a medium containing sorafenib and sunitinib at various concentrations, and cells were incubated at 37°C for 48 h. The drug-containing medium was replaced with a medium containing a WST-8 reagent. After 3 h, absorbance in each well was determined at 450 nm.

### Apoptosis assay

Apoptosis-mediated death of HaCaT cells was examined by a double staining method using an FITC-labeled annexin V/propidium iodide (PI) apoptosis detection kit (BD Biosciences, San Jose, CA, US) according to the manufacturer’s instructions. In brief, cells were washed twice with phosphate-buffered saline (PBS) and then incubated with PBS containing FITC-conjugated Annexin V and PI at 37°C for 30 min. Cells were washed twice with PBS and then incubated with PBS containing 10 µM Hoechst 33258 at 37°C for 30 min. The externalization of phosphatidylserine and permeability to PI were evaluated with an IN Cell Analyzer 2000 (GE Healthcare UK, Ltd., Buckinghamshire, UK). Cells in early apoptosis were positively stained with Annexin V, whereas cells in late apoptosis were positively stained with both Annexin V and PI.

### Plasmid construction

Constitutively active STAT3 (STAT3C) mammalian expression plasmids were kindly provided by Professor Miyajima (University of Tokyo, Tokyo, Japan). STAT3C constructs were transformed into DH-5α-competent cells, and plasmid DNA was extracted using a QIAGEN Plasmid Midi Kit (Qiagen KK, Tokyo, Japan). Extracted plasmids were purified to a grade appropriate for cell culture using phenol and chloroform and stocked at 1 µg/µL in a freezer until use.

### Transient transfection

Transient transfection of cell lines with expression vectors was performed using a Lipofectamine LTX transfection reagent (Life Technologies) according to the manufacturer’s protocol. In brief, cells were grown in 96-well culture plates until they reached ∼80% confluence at a density of 5×10^3^ cells/well or in 60-mm dish at a density of 3×10^5^ cells/dish. The culture medium was replaced with serum-free Opti-MEM (Life Technologies), and cells were transfected with the DNA–lipofectamine complex. HaCaT cells were transiently transfected with 0.1 µg/well of plasmids in 96-well plates or 5 µg/dish of plasmids in 60-mm dish.

### Western blot analysis and immunofluorescence imaging

Western blotting was performed as described previously [18]. Reagent-treated or transfected HaCaT cells were fixed with 4% paraformaldehyde at room temperature for 15 min and then blocked with 5% BSA. Reagent-treated cells were incubated with an anti-phospho-STAT3 antibody, and transfected cells were incubated with an anti-STAT3 antibody, followed by incubation with FITC-conjugated anti-rabbit IgG (Santa Cruz) and visualization with the IN Cell Analyzer 2000.

### Statistical analysis

Statistical analysis was performed with a nonrepeated one-way analysis of variance, followed by the Dunnett’s test for multiple comparisons. A p-value of <0.01 (two-tailed) was considered significant.

## Results

### Effects of STAT3 inhibitor on sorafenib-and sunitinib-induced cell growth inhibition

**Figure 2:**
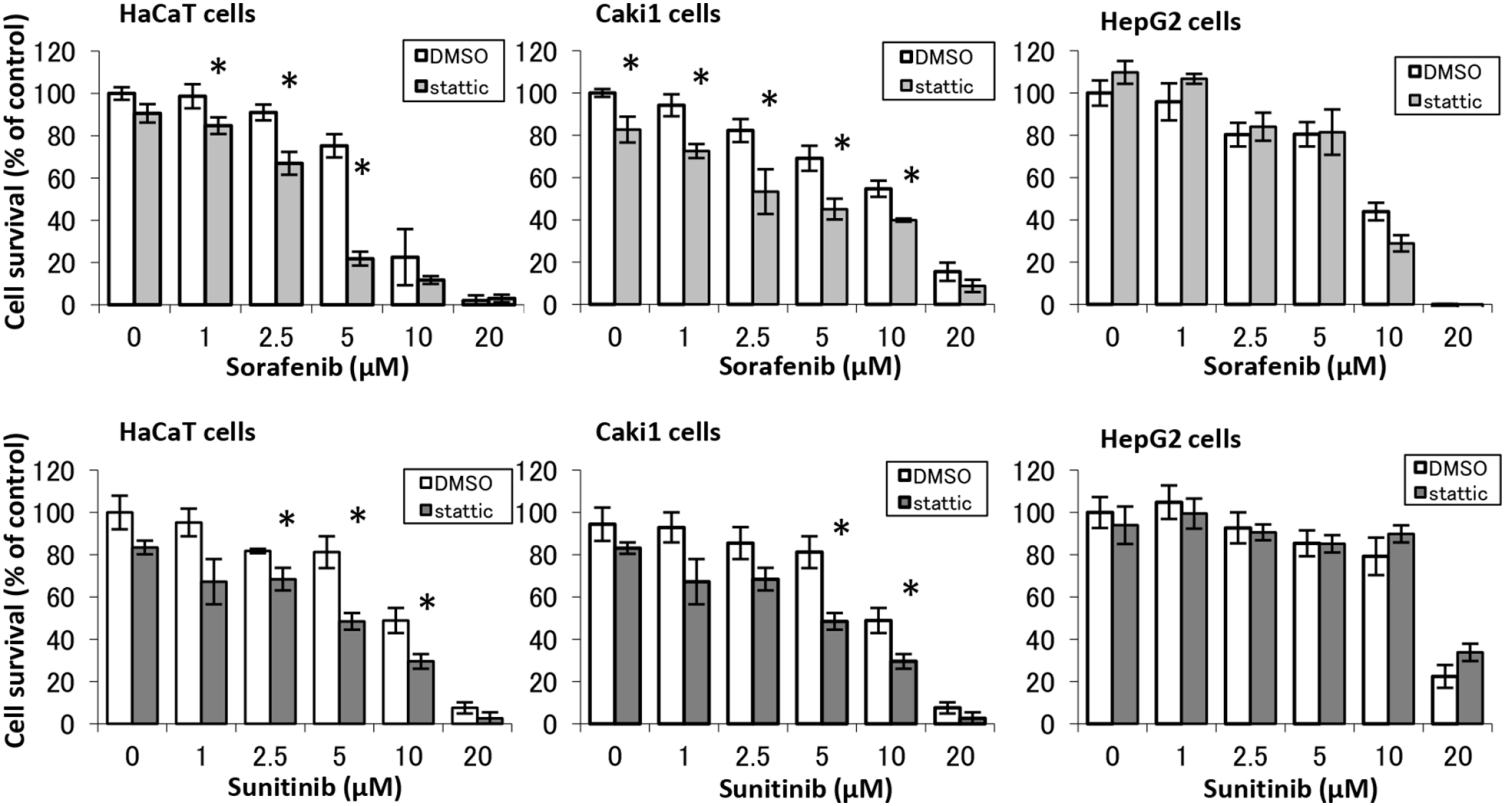
Effects of STAT3 inhibitor on sorafenib- and sunitinib-induced cell growth inhibition. After pretreatment with Stattic (STAT3 inhibitor, 10 µM) or DMSO (solvent) for 20 min, HaCaT, Caki-1, and HepG2 cells were incubated in a medium that included sorafenib or sunitinib at the indicated concentrations for 48 h. Cell viability was determined by WST-8 colorimetric assay. *p<0.01 (Student’s t-test) as compared with control (DMSO). Each bar represents mean ± SD (n = 4).

Figure 2 shows the results for sorafenib- and sunitinib-induced growth inhibition of HaCaT, Caki-1, and HepG2 cells in the pretreatment of the STAT3 inhibitor Stattic. We found that sorafenib- and sunitinib-induced growth inhibition of HaCaT and Caki-1 cells was enhanced by pretreatment with Stattic. In contrast, HepG2 cells were unaffected by pretreatment with Stattic.

### Effects of treatment with sorafenib and sunitinib on STAT3 activity in cells

**Figure 3:**
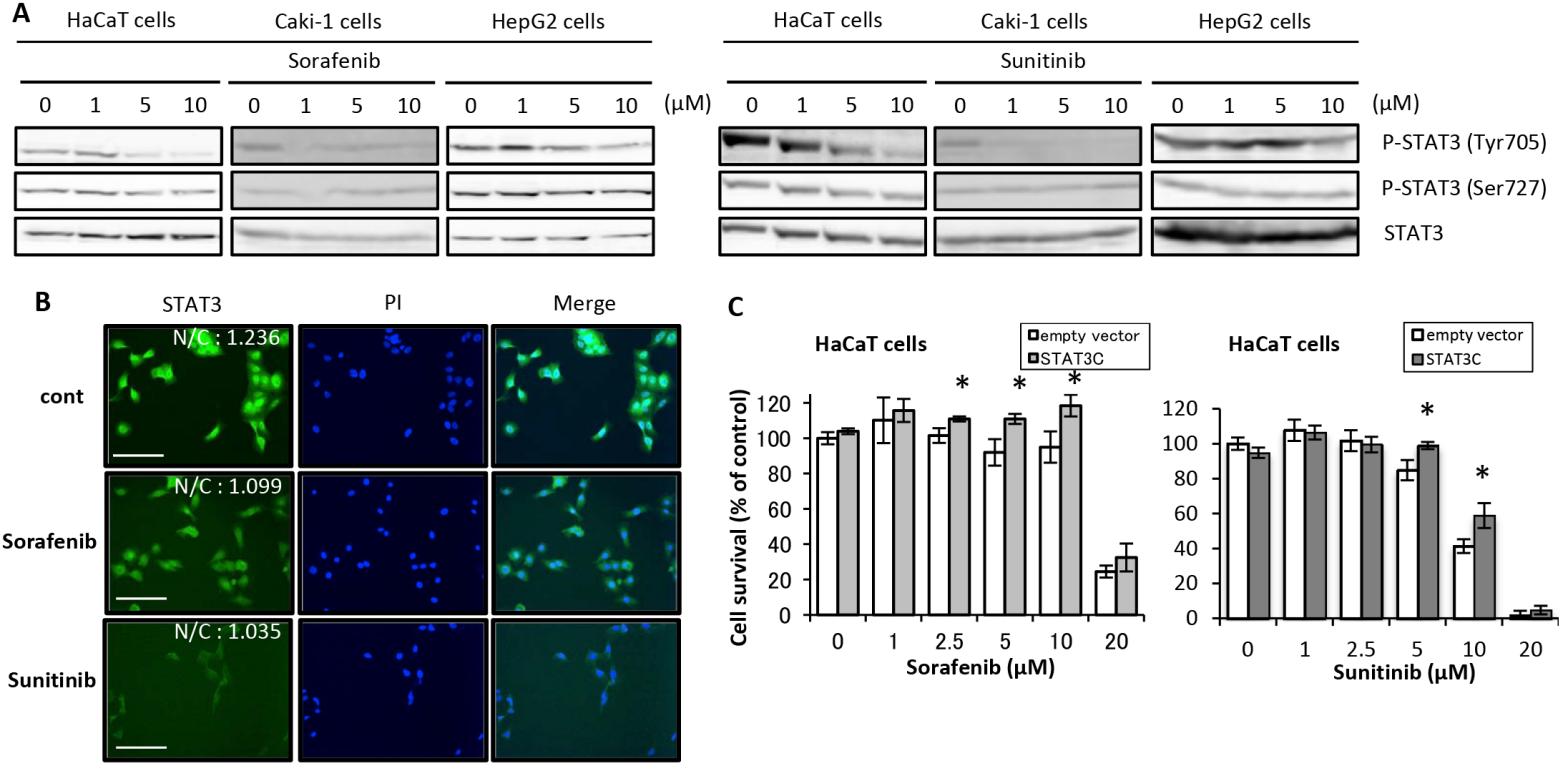
Signal transduction alterations involved in STAT3 after treatment with sorafenib and sunitinib and effects of STAT3C on cytotoxicity of sorafenib and sunitinib. (A) HaCaT, Caki-1, and HepG2 cells were incubated with a medium that included sorafenib or sunitinib at the indicated concentrations for 2 h. Thereafter, Western blot analysis was performed using total cell lysates. (B) Immunostaining images. HaCaT cells were treated with sorafenib (10 µM), sunitinib (10 µM), or DMSO (Control; cont) for 2 h and were fixed and incubated with an anti-STAT3 antibody, followed by incubation with FITC-conjugated anti-rabbit IgG (green), and then visualized with the IN Cell Analyzer 2000. Nuclear translocation of STAT3 was determined with cell population analysis by determining the nucleus/cytoplasm intensity ratio of green fluorescence. Bar shows 50 µm. (C) Effects of STAT3C transfection on sorafenib- and sunitinib-induced cell growth inhibition. HaCaT cells transiently transfected with STAT3C or an empty vector were preincubated for 24 h, followed by incubation in medium containing sorafenib or sunitinib at the indicated concentrations for 48 h. Cell viability was determined by WST-8 colorimetric assay. *p<0.01 (Student’s t-test) as compared with control (DMSO). Each bar represents mean ± SD (n = 4).

STAT3 activity in the presence of sorafenib or sunitinib in each cell line is shown in Figure 3. STAT3 Tyr705 phosphorylation decreased after treatment with sorafenib or sunitinib for 2 h in a dose-dependent manner in HaCaT and Caki-1 cells but not in HepG2 cells. In contrast, STAT3 Ser727 phosphorylation was unaffected (Figure 3A). Moreover, the nuclear translocation of STAT3 decreased after treatment with sorafenib and sunitinib (Figure 3B). Transfecting cells with STAT3C tended to ameliorate the cytotoxicity of sorafenib and sunitinib as compared with transfection with an empty vector (Figure 3C).

### Effects of treatment with sorafenib and sunitinib on apoptosis suppressors and apoptotic effects in HaCaT cells

**Figure 4:**
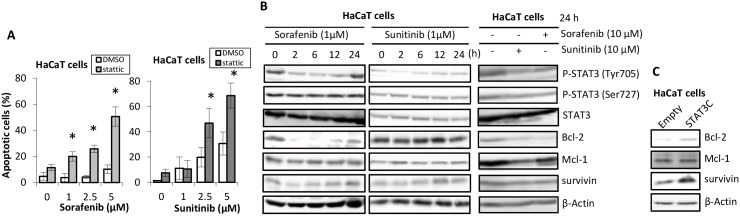
Effects of sorafenib and sunitinib on cellular apoptosis and the expression of apoptosis suppressors. (A) After pretreatment with 10 µM Stattic or DMSO for 20 min, HaCaT cells were incubated in medium containing sorafenib or sunitinib at the indicated concentrations for 24 h. Apoptotic cells were detected using FITC-labeled Annexin V/PI staining with the IN Cell Analyzer 2000. *p<0.01 (Student’s t-test) as compared with control (DMSO). Each bar represents mean ± SD (n = 4). (B) Alterations in STAT3 signal transduction and apoptosis suppressors. HaCaT cells were incubated in medium containing 1 µM sorafenib or sunitinib for the indicated times and with 10 µM sorafenib or sunitinib for 24 h. Western blot analysis was performed using total cell lysates. (C) Alterations of expression of apoptosis suppressors in transfected cells. HaCaT cells transiently transfected with STAT3C or an empty vector were prepared to total cell lysate, and using that persormed western blot analysis.

Proportions of apoptotic cells due to treatment with sorafenib or sunitinib were increased by pretreatment with Stattic (Figure 4A). Moreover, the expression of survivin and bcl-2, apoptosis suppressors whose transcription is regulated by STAT3, decreased after short-term treatment and increased after long-term treatment of HaCaT cells with sorafenib. This was associated with STAT3 Tyr705 phosphorylation (Figure 4B). In contrast, these effects were not found by treatment with sunitinib. After treatment with sorafenib or sunitinib for 24 h, STAT3 Tyr705 phosphorylation and Bcl-2 and Mcl-1 expression decreased, and survivin expression also decreased slightly. Furthermore, in transfecting cells with STAT3C, Bcl-2 and survivin expression increased as compared with empty vector transfecting cells.

### Effects of sorafenib and sunitinib on MAPK activity in HaCaT cells

**Figure 5:**
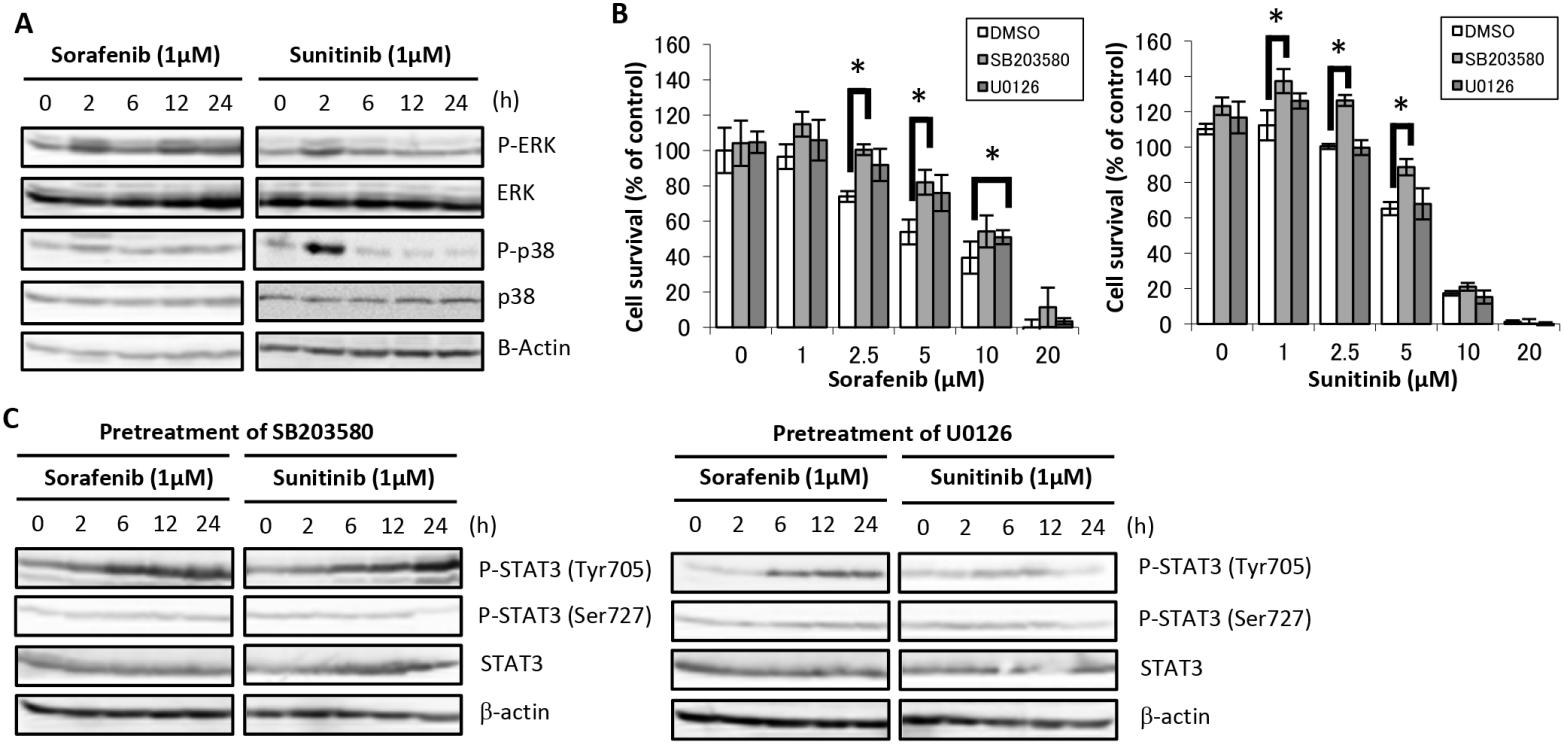
Effects of sorafenib and sunitinib on MAPK activation in HaCaT cells and effects of MAPK inhibitor on sorafenib and sunitinib-induced cell growth inhibition and signal transduction. (A) Alterations in MAPKs signal transduction. HaCaT cells were incubated in medium containing 1 µM sorafenib or sunitinib for the indicated times, followed by Western blot analysis using total cell lysates. (B) Effects of MAPK inhibitor on sorafenib- and sunitinib-induced cell growth inhibition. HaCaT cells were incubated with medium containing sorafenib at the indicated concentrations for 48 h after pretreatment with either U0126 (MEK1/2 inhibitor, 10 µM), SB203580 (p38 MAPK inhibitor, 10 µM), or DMSO (solvent) for 2 h. Cell viability was determined by WST-8 colorimetric assay. *p<0.01 (Student’s t-test) as compared with control (DMSO). Each bar represents mean ± SD (n = 4). (C) Alterations in STAT3 signal transduction in the presence of MAPK inhibitors. HaCaT cells were incubated in medium containing 1 µM sorafenib or sunitinib for the indicated times after pretreatment with 10 µM U0126 for 2 h or 10 µM SB203580 for 2 h, followed by Western blot analysis using total cell lysates.

Figure 5A shows MAPK activity in HaCaT cells after treatment with sorafenib or sunitinib. Treatment with sorafenib induced increased phosphorylation of p38 MAPK and ERK at 2 h, and ERK phosphorylation also increased at 12 and 24 h. Treatment with sunitinib induced the phosphorylation of p38 MAPK at 2 h but not that of ERK. Sorafenib-induced cell growth inhibition was reduced by pretreatment with SB203580 or U0126 in HaCaT cells, whereas it was reduced by pretreatment with SB203580 only (Figure 5B). Figure 5C shows the variations in STAT3 activity induced by sorafenib and sunitinib after pretreatment with the MAPK inhibitors. Pretreatment with SB203580 induced increased STAT3 Tyr705 phosphorylation in a time-dependent manner in cells treated with sorafenib and sunitinib as compared with those treated with sorafenib alone (Figure 4B). In contrast, pretreatment with U0126 induced increased STAT3 Tyr705 phosphorylation in a time-dependent manner in cells treated with sorafenib only.

## Discussion

Pretreatment with Stattic, a small-molecule specific inhibitor of STAT3, synergistically enhanced sorafenib- and sunitinib-induced growth inhibition of HaCaT cells and increased proportions of apoptotic cells (Figures 2 and 4). Furthermore, STAT3C, which dimerized constantly by substituting cysteine residues [19], tended to ameliorate the cytotoxicity of sorafenib and sunitinib. Inhibiting STAT3 activation may strengthen the cytotoxicity of sorafenib and sunitinib for keratinocytes because STAT3 has been reported to be the main factor in the molecular control of cutaneous homeostasis [10], [11]. In our study, because we conducted transient transfection by lipofection method, it was need a high number of cells for survival test with transfected cells. For this reasons, sorafenib and sunitinib-induced cell-growth inhibition in transfected cells was moderate compared with test using non-transfected cells (Figure 2). To clarify the effects of transfection with STAT3C against drug-induced toxicity, it has necessary for study using cells transfected STAT3C stably, and there are issues in the future.

STAT3 Tyr705 phosphorylation decreased after short-term treatment with sorafenib and sunitinib in a dose-dependent manner (Figure 3A), and the nuclear translocation of STAT3 decreased after treatment with sorafenib and sunitinib (Figure 3B). In addition, STAT3 Tyr705 phosphorylation recovered after long-term treatment of HaCaT cells with sorafenib and sunitinib at a low dose (Figure 4B). Thus, sorafenib and sunitinib indirectly interacted with STAT3. So, we suggested that STAT3 is an important factor as also recovering pathway against sorafenib and sunitinib-induced keratinocyte toxicity, and stattic enhanced these toxicities because it inhibited seeking to recover the activity of STAT3 for survival of keratinocytes.

STAT3 has been shown to be required for the development of psoriasis in keratinocytes [20]. In a recent study, psoriasis, a type of epidermal hyperplasia, has been shown to go into remission after treatment with sorafenib [21]. It is believed that sorafenib inhibits STAT3 activity and may inhibit the keratinization of epidermal cells. This hypothesis supports our results and the pathological findings of HFSR as a keratinizing deficiency [6], [22]–[24]. However, to demonstrate this, more detailed studies will be necessary.

Moreover, the cytotoxicity of sorafenib and sunitinib was also enhanced in Caki-1 cells pretreated with Stattic, similar to HaCaT cells. STAT3 Tyr705 phosphorylation in HaCaT and Caki-1 cells decreased after treatment with sorafenib and sunitinib in a dose-dependent manner, whereas this phenomenon was not observed in HepG2 cells (Figure 3A). The efficacy of multikinase inhibitors for RCC has been attributed to its inhibition of STAT3 activity [25], [26]. Based on clinical practice, it is known that the efficacy of molecular-targeted drugs is correlated with their toxicity [27]. Our results support the findings of these reports. We believe that these phenomena in cells can be attributed largely to the effect of STAT3 activity on cell viability, such as in keratinocytes and RCC cells.

STAT3 regulates the expression of Bcl-2, Mcl-1, and survivin, which are apoptosis suppressors [12], [13], [28]. Sorafenib decreased the expression of these apoptosis suppressors by mediating the inhibition of STAT3 activity, which was connected to the enhanced cell growth inhibition by this drug in HaCaT and Caki-1 cells. In particular, survivin has been reported to also be expressed in normal human skin and playing an important role [29], which is in agreement with the concept that nuclear survivin is the main controller of cell division in keratinocytes [30]. In our study, survivin and Bcl-2 expression decreased in cells receiving short-term treatment with sorafenib at a low dose, although their expression recovered with long-term treatment with sorafenib (Figure 4A). In contrast, treatment with sunitinib at 10 µM for 24 h resulted in decreased Bcl-2 and Mcl-1 expression. Moreover, in the cells transfected with STAT3C, ameliorated the cytotoxicity of sorafenib and sunitinib, expression of survivin and Bcl-2 increased significantly (Figure 4C), therefore, it is clear that sorafenib and sunitinib-induced keratinocyte toxicity associate with expression of these apoptosis suppressors. This finding suggests that sorafenib and sunitinib induced apoptosis by depleting apoptosis suppressors mediated by STAT3 inhibition in HaCaT cells, and that sorafenib-induced apoptosis occurred at a lower dose than sunitinib-induced apoptosis (Figures 2 and 4A, B).

STAT3 activity is negatively regulated by MAPK pathways [15], [31]. Moreover, epidermal hyperplasia caused by Raf-MAPK requires the interaction of STAT3 signaling, as STAT3 activity involved in MAPK pathways is important for keratinocyte proliferation and differentiation [32]. Our results show that sorafenib and sunitinib induced alterations in MAPK activity, and that MAPK inhibitors partially ameliorated the toxicity of sorafenib and sunitinib for HaCaT cells (Figure 5). Sorafenib inhibits cell proliferation by blocking MAPK pathways [3], [33], [34]. Based on our results with HaCaT cells, low-dose sorafenib induced ERK phosphorylation. It is possible that sorafenib has a low affinity for MEK in keratinocytes based on our results that sorafenib induced increased ERK phosphorylation in HaCaT cells in a time- and dose-dependent manner but had the opposite effects in Caki-1 and HepG2 cells (data not shown). Additional investigations will be necessary to clarify these phenomena.

Sorafenib and sunitinib also induce the generation of reactive oxygen species (ROS) in various cell types [35]–[37]. Because p38 MAPK is activated by oxidative stress, sorafenib and sunitinib may inhibit STAT3 activity via negative regulation by p38 MAPK, and p38 MAPK inhibitor can ameliorate the cytotoxicity of sorafenib and sunitinib. In a recent clinical report, skin reaction due to molecular-targeted therapy with multikinase inhibitors was triggered by the physical pressure from walking, hand washing, or other daily activities [6], [38], [39]. Our study results support the finding of this clinical report because physical stress also activates p38 MAPK.

In conclusion, we demonstrated that the toxicity of sorafenib and sunitinib for keratinocytes was induced by decreased apoptosis suppressors via the inhibition of STAT3 activity. The dermatological side effects of malti kinase inhibitors are often accompanied by pain, therefore, these skin reactions may cause performance impairment of fingers or gait disorder, leading to a decreased quality of life. Current strategies for the cutaneous adverse events are symptomatic therapy based on clinical experience. Therefor, novel strategies has been advocated to control pathogenic mechanisms of the toxic effects on the skin.
